# pH-responsive hydrogel with dual-crosslinked network of polyvinyl alcohol/boric acid for controlled release of salvianolic acid B: novel pro-regenerative mechanisms in scar inhibition and wound healing

**DOI:** 10.1093/rb/rbaf002

**Published:** 2025-01-02

**Authors:** Wei Song, Chao Zhang, Zhao Li, Kejia Li, Yi Kong, Jinpeng Du, Yue Kong, Xu Guo, Xiaoyan Ju, Meng Zhu, Ye Tian, Sha Huang, Zhongwei Niu

**Affiliations:** Key Laboratory of Photochemical Conversion and Optoelectronic Materials, Technical Institute of Physics and Chemistry, Chinese Academy of Sciences, Beijing 100190, China; University of Chinese Academy of Sciences, Beijing 100049, China; Medical Innovation Research Department, Research Center for Wound Repair and Tissue Regeneration, Chinese PLA General Hospital, Beijing 100048, China; Medical Innovation Research Department, Research Center for Wound Repair and Tissue Regeneration, Chinese PLA General Hospital, Beijing 100048, China; School of Medicine, Nankai University, Tianjin 300071, China; Medical Innovation Research Department, Research Center for Wound Repair and Tissue Regeneration, Chinese PLA General Hospital, Beijing 100048, China; Key Laboratory of Photochemical Conversion and Optoelectronic Materials, Technical Institute of Physics and Chemistry, Chinese Academy of Sciences, Beijing 100190, China; University of Chinese Academy of Sciences, Beijing 100049, China; Medical Innovation Research Department, Research Center for Wound Repair and Tissue Regeneration, Chinese PLA General Hospital, Beijing 100048, China; Medical Innovation Research Department, Research Center for Wound Repair and Tissue Regeneration, Chinese PLA General Hospital, Beijing 100048, China; Medical Innovation Research Department, Research Center for Wound Repair and Tissue Regeneration, Chinese PLA General Hospital, Beijing 100048, China; Medical Innovation Research Department, Research Center for Wound Repair and Tissue Regeneration, Chinese PLA General Hospital, Beijing 100048, China; Key Laboratory of Photochemical Conversion and Optoelectronic Materials, Technical Institute of Physics and Chemistry, Chinese Academy of Sciences, Beijing 100190, China; Key Laboratory of Photochemical Conversion and Optoelectronic Materials, Technical Institute of Physics and Chemistry, Chinese Academy of Sciences, Beijing 100190, China; Key Laboratory of Photochemical Conversion and Optoelectronic Materials, Technical Institute of Physics and Chemistry, Chinese Academy of Sciences, Beijing 100190, China; Medical Innovation Research Department, Research Center for Wound Repair and Tissue Regeneration, Chinese PLA General Hospital, Beijing 100048, China; Key Laboratory of Photochemical Conversion and Optoelectronic Materials, Technical Institute of Physics and Chemistry, Chinese Academy of Sciences, Beijing 100190, China; School of Future Technology, University of Chinese Academy of Sciences, Beijing 100049, China

**Keywords:** controlled release, salvianolic acid B, scar inhibition, skin regeneration, dual-crosslinked network

## Abstract

This study investigates a novel pH-responsive hydrogel composed of polyvinyl alcohol (PVA) and boric acid (BA) designed for the controlled release of salvianolic acid B (SAB), addressing the critical challenge of scar formation and skin regeneration. The dual-crosslinked network architecture of the hydrogel exhibits remarkable pH sensitivity, enabling it to achieve a peak SAB release within 48 hours in the acidic microenvironment characteristic of early-stage wound healing. *In vitro* assessments demonstrated that the PVA-BA-SAB hydrogel significantly inhibits fibroblast activation and mitigates abnormal collagen deposition, effectively preventing excessive scar formation. Transcriptome sequencing reveals the potential role of PVA-BA-SAB hydrogel in balancing TGF-β and Wnt signaling pathways. Furthermore, *in vivo* studies revealed enhanced tissue regeneration, characterized by improved collagen organization and increased vascularization, as well as the promotion of mature hair follicle development. The hydrogel’s biocompatibility, mechanical robustness and adhesive properties were also thoroughly evaluated, confirming its suitability for clinical applications. These findings suggest that the PVA-BA-SAB hydrogel fully exerts the excellent characteristics of biomaterials and maximizes the pharmacological effect of SAB. Our innovative drug delivery system not only facilitates enhanced wound healing but also offers a strategic approach to minimize scarring. This research provides valuable insights into innovative therapeutic strategies for effective wound management and tissue repair.

## Graphical abstract

**Figure:**
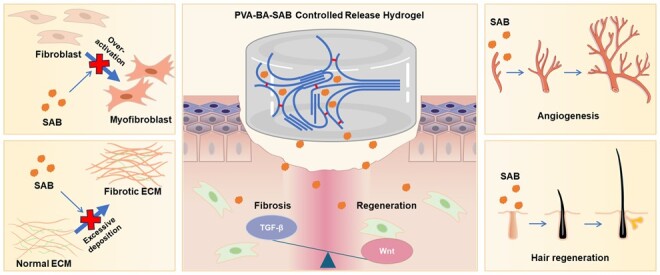

## Introduction

Scarring is an inevitable outcome of the skin’s repair process following damage from burns, scalds, traumatic injuries, surgical interventions and various other forms of injury [1, 2]. This phenomenon significantly affects both the physical and psychological well-being of patients, often leading to complications that extend beyond the visible scars themselves [3]. Traditional treatments for scars can be costly and frequently yield suboptimal results, with some options introducing potential side effects that further complicate patient care [4, 5]. Scar formation primarily arises from the persistent and excessive activation of fibroblasts, coupled with abnormal collagen deposition [6, 7]. Rather than addressing scars after formation, a more promising approach lies in modulating fibroblast behavior during the wound healing process to prevent scar development and promote skin regeneration altogether [8, 9].

Pharmacological interventions offer a viable pathway for scar prevention; however, the repertoire of effective drugs remains limited [5, 10]. Furthermore, the mechanisms underlying many available treatments are not well understood, which raises concerns regarding their safety and efficacy. Among the emerging therapeutic agents, salvianolic acid B (SAB), a bioactive compound derived from *Salvia divinorum*, has gained attention for its potential in scar management [11]. SAB has demonstrated the ability to inhibit both the proliferation and migration of human skin fibroblasts (HSFs) *in vitro*, suggesting its efficacy in mitigating hypertrophic scar formation [12, 13]. Additionally, SAB exhibits notable anti-inflammatory and anti-fibrotic properties, as evidenced by studies in pulmonary fibrosis models, indicating its capacity to regulate excessive extracellular matrix (ECM) deposition [14, 15]. Despite these promising attributes, the clinical utility of SAB is limited by its short biological half-life, which results in rapid metabolism and necessitates frequent administration at higher dosages when applied topically [16, 17].

To address these challenges, we developed a novel pH-responsive drug delivery system utilizing a hydrogel with dual-crosslinked network that integrates physical microcrystalline regions of polyvinyl alcohol (PVA) with chemical boronic ester bonds formed between PVA and boric acid (BA). PVA is a well-characterized, water-soluble polymer recognized for its biocompatibility and capacity to form hydrogels through freezing and thawing cycles [18]. However, the mechanical properties of PVA hydrogels can be inadequate for certain applications [19]. By incorporating BA, we create a dynamic crosslinked network through boronic ester bonds, allowing for tunable enhancements in the hydrogel’s mechanical characteristics [20]. More importantly, the pH-responsive nature of the boronic ester bonds enables the hydrogel to intelligently release drugs in the slightly acidic microenvironment typical of wounds [21, 22].

The integration of SAB into this PVA-BA hydrogel establishes a controlled-release system that prolongs the residence time of SAB at the wound site while ensuring a steady and uniform drug release. The dual-crosslinked network of the hydrogel provides both physical and chemical crosslinking, improving its mechanical strength and stability—essential qualities for enduring the dynamic conditions of a wound environment and facilitating effective drug delivery. Remarkably, delivering SAB via the PVA-BA hydrogel not only demonstrates anti-fibrotic effects, such as inhibiting fibroblast activation and optimizing collagen deposition, but also significantly enhances the regeneration of the epidermis, blood vessels and hair follicles. Transcriptome sequencing indicates that these effects are mediated through the inhibition of the TGF-β pathway and the activation of the Wnt signaling pathway. This controlled-release system successfully optimizes the local therapeutic efficacy of SAB while minimizing potential systemic side effects.

## Materials and methods

### Cell culture and treatment

Human skin fibroblast cell line (iCell) was cultured in Dulbecco’s Modified Eagle Medium (DMEM) supplemented with 10% Fetal Bovine Serum (FBS) and 1% P/S at 37°C with 5% CO_2_. Passages 5–15 were used for the following experiment. To activate HSFs, recombinant human TGF-β1 (CA59, Novoprotein, China) with a final concentration of 20 ng/ml was added after HSF inoculation and adhesion.

### Preparation of PVA-BA-SAB hydrogels

To prepare PVA-BA-SAB hydrogel, aqueous solutions of 6% (w/v) PVA (041243-100 g, Alfa Aesar, China) and 1% (w/v) BA (Beijing Chemical Factory, China) were prepared by mixing and stirring in equal volumes. An SAB (SS8100, Solarbio, China) solution with a final concentration of 100 μg/ml was then added after the mixture of PVA and BA. The solutions were then incubated at −20°C overnight. The following day, the mixture was removed and melted at room temperature. This freeze–thaw cycle was repeated 3 times.

### Crosslinking mechanisms of PVA-BA-SAB hydrogels

#### X-ray diffraction

X-ray diffraction (XRD, Rigaku Ultima IV, Japan) was employed to substantiate the formation of a microcrystalline zone in the hydrogels. The test was conducted with the following parameters: wavelength of 1.5418, voltage of 40 kV, current of 40 mA, scanning speed of 2°/min and a test range of 2*θ* of 5–90°.

#### Fourier transform infrared

The formation of boronic ester bonds was detected using a Fourier Transform Infrared Spectrometer (Thermo Scientific Nicolet iS20, USA). Once the hydrogel had been frozen and dried, it was placed in a mortar and mixed with potassium bromide powder, then ground until a fine powder was formed. Subsequently, the powder was compressed and placed in an apparatus for the detection of infrared spectra.

#### H nuclear magnetic resonance

The binding mechanism of SAB in PVA-BA-SAB hydrogel was explored by H Nuclear Magnetic Resonance (NMR-H). An amount of 8.0 mg of SAB standard was weighed and dissolved in 1.2 ml DMSO-d_6_ (Energy, China). Then the solution was sonicated at 55°C to dissolve. A volume of 300 μl DMSO-d_6_ SAB solution was added to 3 NMR tubes. An amount of 3.0 mg PVA and BA powder were weighed separately, dissolved in 450 µl deuterated DMSO (Energy, China). A volume of 300 µl deuterated DMSO, DMSO-d_6_ PVA and DMSO-d_6_ BA were added to each of the 3 NMR tubes. After mixing, the samples were returned to room temperature and the hydrogen spectra of the samples were measured using a 600 M NMR spectrometer (Bruker Ascend 600).

### Basic properties of PVA-BA-SAB hydrogels

#### Microstructure and pore size

Scanning electron microscopy (SEM) was used to determine the microstructural features and pore size of the hydrogels. The prepared hydrogels were rapidly frozen in liquid nitrogen and then freeze-dried. A cross-section of the sample was sprayed with gold and imaged by SEM (TESCAN MIRA LMS, Czech Republic). The pore size was subsequently measured using Image J software (National Institutes of Health, Bethesda, Virginia, USA).

#### Biocompatibility

The HSF was seeded on the coverslips placed in the lower side of the 24-well transwell chamber at a density of 5 × 10^4^ cells per well in 500 μl culture medium and incubated at 37°C in a 5% CO_2_ atmosphere. After cell adhesion and pre-activation, PVA-BA or PVA-BA-SAB hydrogels were added on the upper side of the transwell chamber for 48 hours. Then, live/dead staining (L3224, Invitrogen) was employed to assess survival of cells. Images were obtained through a confocal microscope (Leica, SP8 FALCON).

#### Rheological behavior

Rheological testing of the PVA-BA-SAB hydrogel was conducted using a rheometer (Anton Paar MCR 302, Austria). The frequency was from 0.1 to 100 rad/s, with a constant strain of 0.1%. Viscosity tests were conducted at shear rates ranging from 0.01 to 100 rad/s.

### Cumulative release of SAB from PVA-BA-SAB hydrogel

The release behavior of SAB from PVA-BA-SAB hydrogel was determined by high-performance liquid chromatography (HPLC, Shimadzu 20A). PVA-BA-SAB hydrogel was introduced into 10 ml PBS (pH = 5.5 or 7.4) at 37°C. At designated time points (2, 4, 6, 8, 10, 12, 24, 36 and 48 hours), 500 μl supernatant was collected and replaced with an equal volume of fresh PBS. Standard SAB solutions were prepared at concentrations of 5, 10, 15, 20 and 40 μg/ml. Then standard concentration–absorbance curves were obtained by HPLC. The SAB concentration released from the samples at each time point was calculated via the standard curve, and the percentage of released salvinorin B was accumulated. The percentage of SAB release was calculated using the following formula:

Cumulative release of SAB (%)=(released SAB/total SAB)×100%

### Cell migration

The HSFs were seeded in 6-well plates at a density of 2 × 10^5^ per well, while the concentration of FBS in the medium was adjusted to 1% to inhibit proliferation. After cell adhesion, TGF-β1 was used to activate HSFs. Once 90% confluence was attained, the cells were scratched perpendicularly with a pipette tip and rinsed with PBS three times. Subsequently, for the treatment group, PVA-BA-SAB hydrogel were added on the upper side of the 6-well transwell chamber for 48 hours. The closure of the scratches was recorded by optical microscopy at 0, 24 and 48 hours. Then, 5 view fields were randomly picked for observation under an optical microscope (DMI4000B, Leica, Frankfurt, Germany). The relative area was quantified and calculated using ImageJ software. The area at 0 h was recorded as *S*0, and the area at 24 hours or 48 hours was recorded as *S*t. Cell migration was calculated using the following formula:

Cell migration (%)=(S0−St)/S0×100%

### RT-qPCR

Pre-activated HSF, PVA-BA-treated HSF and PVA-BA-SAB-treated HSF were collected with Trizol reagent (Invitrogen, USA). The isolation of total RNA from cells was following the manufacturer’s protocol. RNA concentration was determined by using a NanoPhotometer (Implen GmbH, P-330-31, Germany). Reverse transcription involved use of a complementary DNA synthesis kit (Takara, China). Gene expression was analyzed quantitatively by using SYBR green with the 7500 Real-Time PCR System (Takara, China). Primers and probes for genes were designed based on published gene sequences (National Center for Biotechnology Information and PubMed). The expression of each gene was normalized to that for glyceraldehyde-3-phosphate dehydrogenase and analyzed by the 2^−△△CT^ method. Each sample was assessed in triplicate. The specific gene primers used for qPCR analysis can be found in Supplementary Table S1.

### Immunofluorescence

Cells with different treatment were first fixed with 4% paraformaldehyde for a duration of 30 minutes. The samples were washed with PBS and then permeabilized and blocked using a solution containing 0.1% Triton X-100 in 5% BSA. Subsequently, the samples were incubated with the primary antibody, with the incubation taking place in a wet box at 4°C for overnight. After the primary antibody incubation, the samples were washed five times with PBS to remove any unbound antibodies. Next, the samples were incubated with the appropriate secondary antibodies obtained from Abcam for a duration of 1 hour. Following another round of washing with PBS, the nuclei of the samples were labeled with DAPI. Finally, the fluorescent signals emitted by the samples were detected and visualized using a confocal microscopy system (SP8, Leica, Frankfurt, Germany). The antibodies used were listed in Supplementary Table S2.

### Transcriptome sequencing

The transcriptome of the pre-activated HSF and PVA-BA-SAB-treated HSF was sequenced using the Illumina NovaSeqTM 6000 platform by LC Sciences. Differentially expressed genes (DEGs) were then subjected to enrichment analysis of Gene Ontology (GO) functions and KEGG pathways. All DEGs were mapped to GO terms in the Gene Ontology database (http://www.geneontology.org/), gene numbers were calculated for every term, and significantly enriched GO terms in DEGs compared to the genome background were defined by hypergeometric test. KEGG is the major public pathway-related database. Principal component analysis (PCA), dot plot, heatmap were plotted by https://www.bioinformatics.com.cn, an online platform for data analysis and visualization.

### Construction of skin wound model on mice

All the animal experiments were performed according to the guidelines established by Institutional Animal Care and Use Committee of Chinese PLA General Hospital and approved by the Animal Ethics Committee of the Chinese PLA General Hospital (approval no. S2020-407-01). C57BL/6 mice 6–8 weeks of age were purchased from SPF Biotechnology Limited (Beijing, China). All mice were housed at 23 ± 3°C with a 12/12-light/dark cycle and received lab chow and water *ad libitum*.

Mice were weighed and anesthetized via intraperitoneal injection of 20 g/l sodium pentobarbital at 50 mg/kg. A 1 cm circular full-layer skin wound was constructed in the center of the back on each mouse. The mice were randomly assigned to four groups: a blank control group (Control), a simple SAB treating group (SAB), a PVA-BA hydrogel treating group (PVA-BA), and a PVA-BA-SAB hydrogel treating group (PVA-BA-SAB). Each group consisted of 9 mice. After appropriate treatment, the wound was covered by external dressings and bandaged for a period of 21 days. Wound healing was assessed by imaging on days 0, 7, 14, 21. Wound closure was analyzed by comparing the relative wound size (*S*a) at a given time to 0d-post-wounding size (*S*n) using the ImageJ software. Wound healing rate was calculated using the following formula:

Wound healing rate (%)=(Sa—Sn)/Sa×100%

### Histopathological observation

On day 21, the dorsal wound tissues within 0.5 cm of the wound edge were collected. The samples were fixed with 4% paraformaldehyde and embedded in paraffin in accordance with standard procedures. To evaluate the quality of wound healing following different treatments, HE staining, Matson’s trichrome staining, Sirius red staining, and immunofluorescence staining were performed. A digital panoramic scanner (3D Histech) was employed to sweep the sections stained with HE. A fully automated digital slide scanning system (Zeiss) was used for Masson’s trichrome staining and immunofluorescence staining. The slide scanning system (Zeiss) was employed for Sirius red staining and processed on the ZEISS software for observation and screenshots. Quantitative statistics and analysis of images selected were performed using Image J software.

### Statistical analysis

The statistical analysis was conducted using GraphPad Prism 8.0 software. The data were expressed as mean ± standard deviation (SD). To assess differences between multiple groups, an ANOVA and Tukey’s multiple comparison test were employed. Differences between two groups were analyzed by t-test. In all experiments, a significance level of *P *<* *0.05 was considered statistically significant.

## Results

### Preparation and crosslinking mechanism of PVA-BA-SAB hydrogel

**Figure 1.**
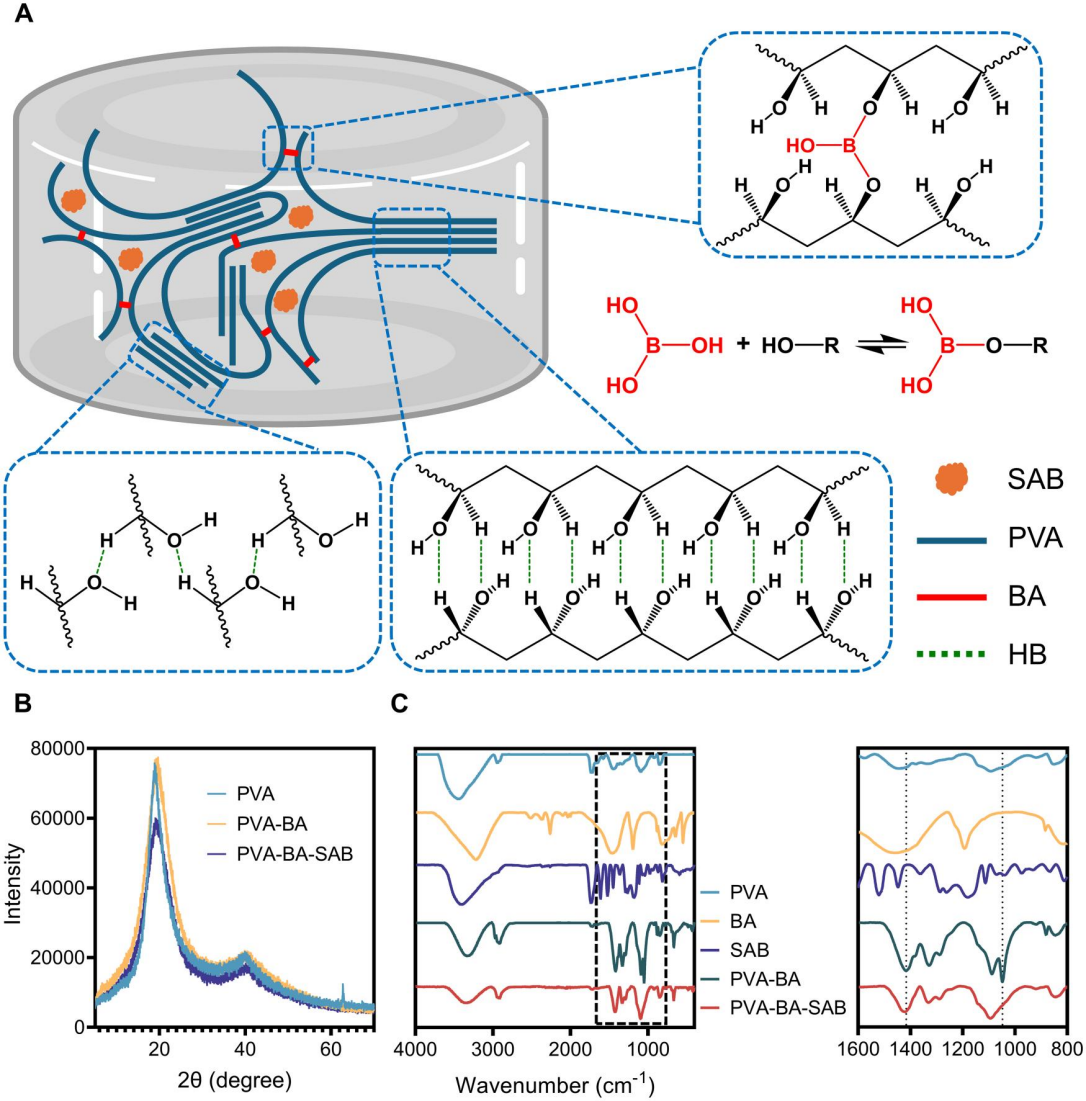
Preparation and crosslinking mechanism of PVA-BA-SAB hydrogel. (**A**) Schematic diagram of PVABA-SAB hydrogel formation. (**B**) X-ray diffraction of PVA, PVA-BA hydrogel and PVA-BA-SAB hydrogel. (**C**) Fourier transform infrared spectroscopy of PVA, BA, SAB, PVA-BA hydrogel and PVA-BA-SAB hydrogel.

The schematic illustration for the fabrication of PVA-BA-SAB hydrogel is shown in Figure 1A. The physical–chemical dual-crosslinked network in the PVA-BA-SAB hydrogel and the binding mode of SAB are first confirmed. The XRD of the PVA, PVA-BA and PVA-BA-SAB hydrogels is shown in Figure 1B. It showed that a sharp signal peak is formed at around 20°, representing the physical microcrystalline zones formed by freeze–thaw of PVA. Meanwhile, the addition of BA and SAB had no effect on the formation of the physical crosslinking network. The interaction of the components within the hydrogel was detected by FTIR (Figure 1C). The telescopic vibrational peaks representing C–O and B–O in B–O–C at 1048 and 1416 cm^−1^ indicated the formation of boronic ester bonds between PVA and BA in the hydrogel, and no new peaks were observed after adding SAB. These results proved the existence of the physical crosslinking network formed by PVA and the chemical crosslinking network between PBA and BA in the PBA-BA-SAB hydrogel.

NMR was employed to investigate the loading mode of SAB in the PVA-BA-SAB hydrogel (Supplementary Figure S1). The NMR spectra demonstrated that the peak of SAB remained relatively stationary in different environments, while the position of the water peak of the solvent was observed at 3.33. The addition of SAB resulted in a slight shift to the left and broadening of this peak, while the introduction of PVA or BA led to a more pronounced shift and significant broadening. This indicates that there is no robust hydrogen bonding between SAB and BA and PVA. Instead, all three substances preferentially form hydrogen bonds with water molecules.

### Basic properties of PVA-BA-SAB hydrogel

**Figure 2.**
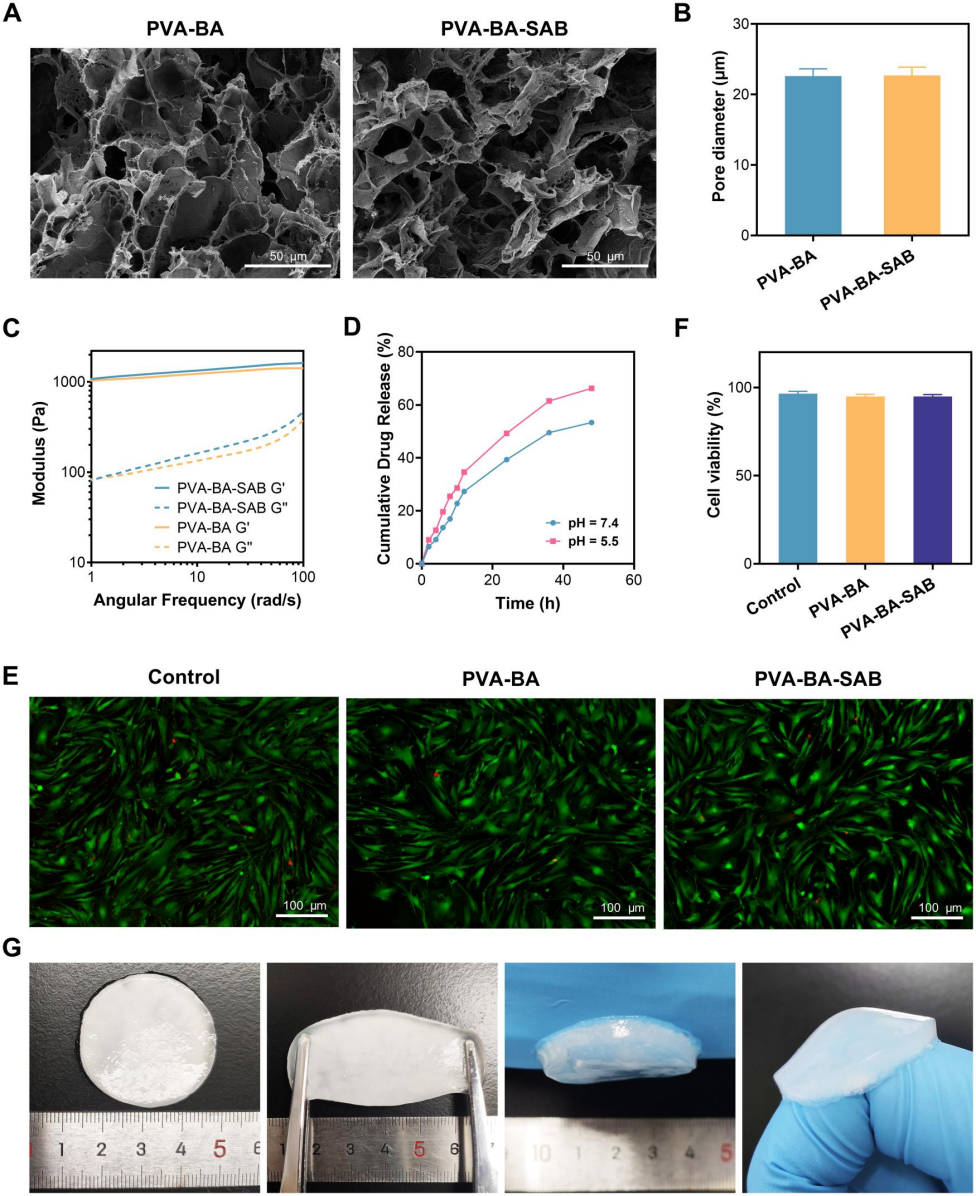
Basic properties of PVA-BA-SAB hydrogel. (**A**) SEM images of PVA-BA and PVA-BA-SAB hydrogels. (**B**) Pore sizes of PVA-BA and PVA-BA-SAB hydrogels. (**C**) Modulus-frequency curves of PVA-BA and PVA-BASAB hydrogels. (**D**) Cumulative release of SAB from PVA-BA-SAB hydrogel responded to acidity of microenvironment. (**E**) Live/dead staining images of PVA-BA and PVA-BA-SAB hydrogels. (**F**) Cell viability of PVA-BA and PVA-BA-SAB hydrogels. (**G**) Photographs of original PVA-BA-SAB hydrogel, stretched hydrogel and adhered hydrogel.

The microstructure of the PVA-BA-SAB hydrogel was observed by SEM (Figure 2A). The hydrogel presented a uniform porous structure. The incorporation of SAB did not affect the structural integrity of the dual-crosslinked network. The porosities of PVA-BA and PVA-BA-SAB hydrogels were also similar (Figure 2B). It further suggested that SAB did not participate in the formation of the network but rather dispersed within it, potentially through hydrogen bonding or other means. The energy storage modulus of the hydrogel was consistently greater than the energy dissipation modulus in the rheological test (Figure 2C), indicating that the hydrogel exhibited optimal gel-forming behavior. The cumulative percentage of released SAB from the PVA-BA-SAB hydrogel was then evaluated by HPLC (Figure 2D) to ascertain the pH-responsive controlled release property. The concentration of SAB was calculated from the standard curve and expressed as a cumulative percentage. The findings indicated that SAB did not experience an initial burst release, and ∼53.34% of SAB was released gradually from the hydrogel with pH 7.4 within 48 hours. When the hydrogel was placed in an acidic environment with pH 5.5, the amount of SAB released rises to about 66.25%, certifying the pH-responsive character dominated by boronic ester bonds. The biocompatibility of PVA-BA-SAB was tested by Live/Dead staining (Figure 2E and F). There was no significant difference in the cell viability of the PVA-BA and PVA-BA-SAB hydrogels compared to the control group, indicating excellent biocompatibility. The macroscopic properties of the PVA-BA-SAB hydrogel were observed in the next step (Figure 2G). The hydrogel exhibited limited transparency, softness, and ease of stretchability. It was soft and easily stretchable. Excellent adhesion ensured that it can be completely attached to the skin surface and remain adhered even at joints. In conclusion, the PVA-BA-SAB hydrogel showed stability, safety, adhesion and drug controlled-release ability, which are essential for a drug-loading dressing.

### Effect of PVA-BA-SAB hydrogel on the biological behavior of fibroblasts

**Figure 3.**
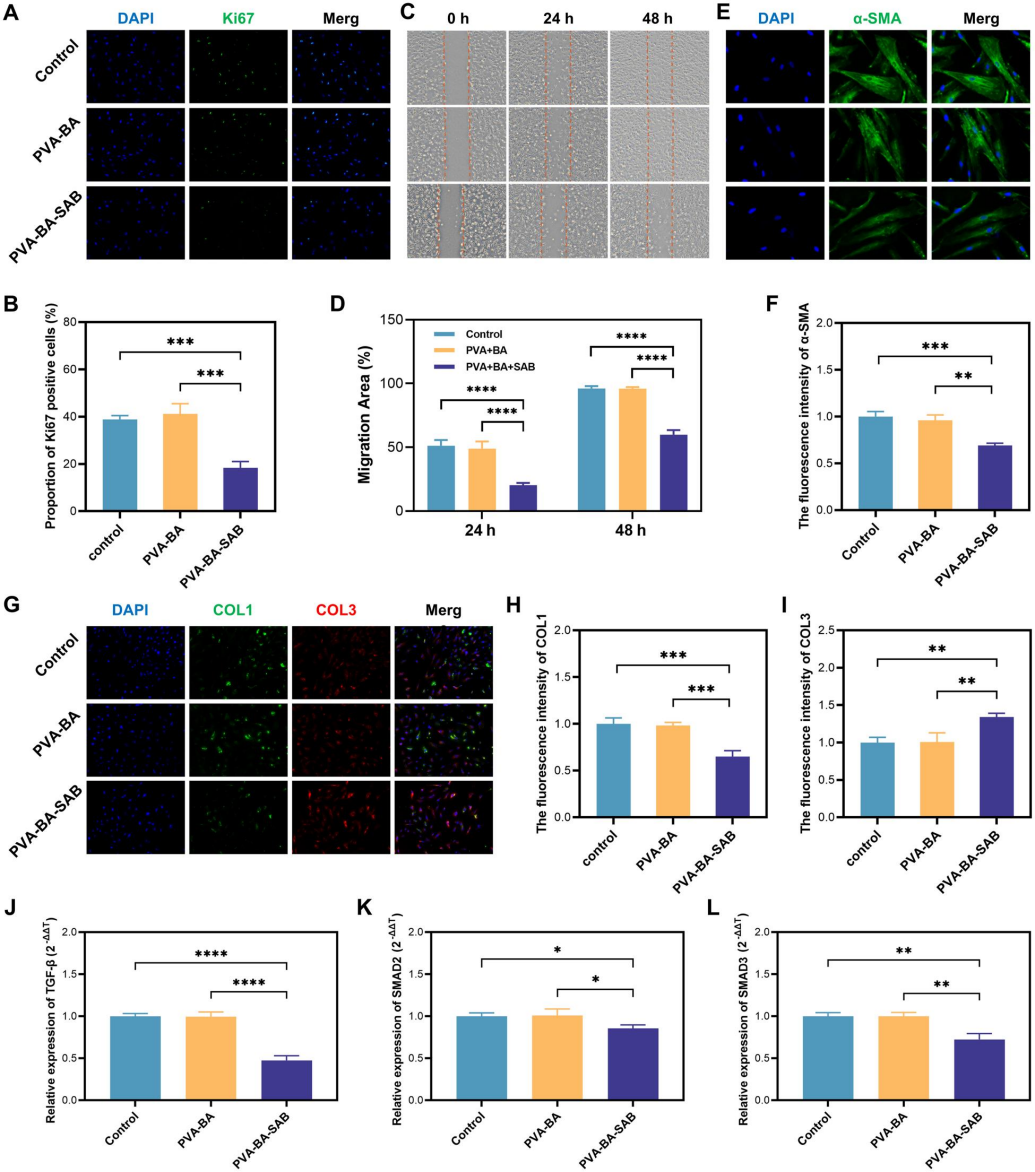
Effect of PVA-BA-SAB hydrogel on the biological behavior of fibroblasts. (**A**) Immunofluorescence staining of Ki67. (**B**) The fluorescence intensity of Ki67. (**C**) Representative images of the gap areas at 0, 24 and 48 hours in scratch wound healing assay. (**D**) Quantification of the percentage of the remaining area to the initial scratch area. (**E**) Immunofluorescence staining of α-SMA. (**F**) The fluorescence intensity of α-SMA. (**G**) Immunofluorescence staining of COL1 and COL3. (**H**) The fluorescence intensity of COL1. (**I**) The fluorescence intensity of COL3. (**J–L**) Transcriptional expression of TGF-β, SMAD2 and SMAD3 evaluated by RT qPCR. *P < 0.05, **P < 0.01, ***P < 0.001 and ****P < 0.0001.

To restore the temporary activation of fibroblasts, which are the principal functional cells when hydrogels are applied to wounds, we pre-activated HSF using TGF-β. The effect of PVA-BA-SAB hydrogel on HSF proliferation was evaluated by immunofluorescence staining for the proliferation marker Ki67 (Figure 3A and B). No significant differences were observed in the PVA-BA hydrogel compared to the control group, while the proportion of Ki67 positive cells was significantly decreased when treated by PVA-BA-SAB hydrogel. Subsequently, the impact of the PVA-BA-SAB hydrogel on HSF migration was investigated through cell wound scratch assay (Figure 3C and D). After 24- or 48-hour post-scratching, the migration rate of HSF was significantly reduced in the PVA-BA-SAB group relative to the control group. No significant difference was observed in the migration rate in the PVA-BA group. It also highlighted the inhibitory effect of SAB on HSF migration.

The impact of the PVA-BA-SAB hydrogel on HSF activation was evaluated by the myofibroblast marker α-SMA (Figure 3E and F). The PVA-BA-SAB hydrogel was observed to exert a substantial inhibitory effect on the activation of HSF. The changes in HSF secretory function before and after PVA-BA-SAB hydrogel treatment were further tested (Figure 3G–I). It demonstrated that the PVA-BA-SAB hydrogel was effective in reversing the level of type I collagen secretion. In contrast, the secretion of type III collagen improved. The expression of these related markers at the mRNA level was further confirmed by RT-qPCR, and the results were similar (Supplementary Figure S2). To elucidate the anti-activation mechanism of PVA-BA-SAB hydrogel, we detected the expression level of the TGF-β/SMAD signaling pathway (Figure 3J–L), a key pathway for fibroblast activation. Treatment of PVA-BA-SAB hydrogel significantly reduced the expression of these genes.

### Mechanisms of PVA-BA-SAB hydrogel in inhibiting fibrosis and promoting regeneration

**Figure 4.**
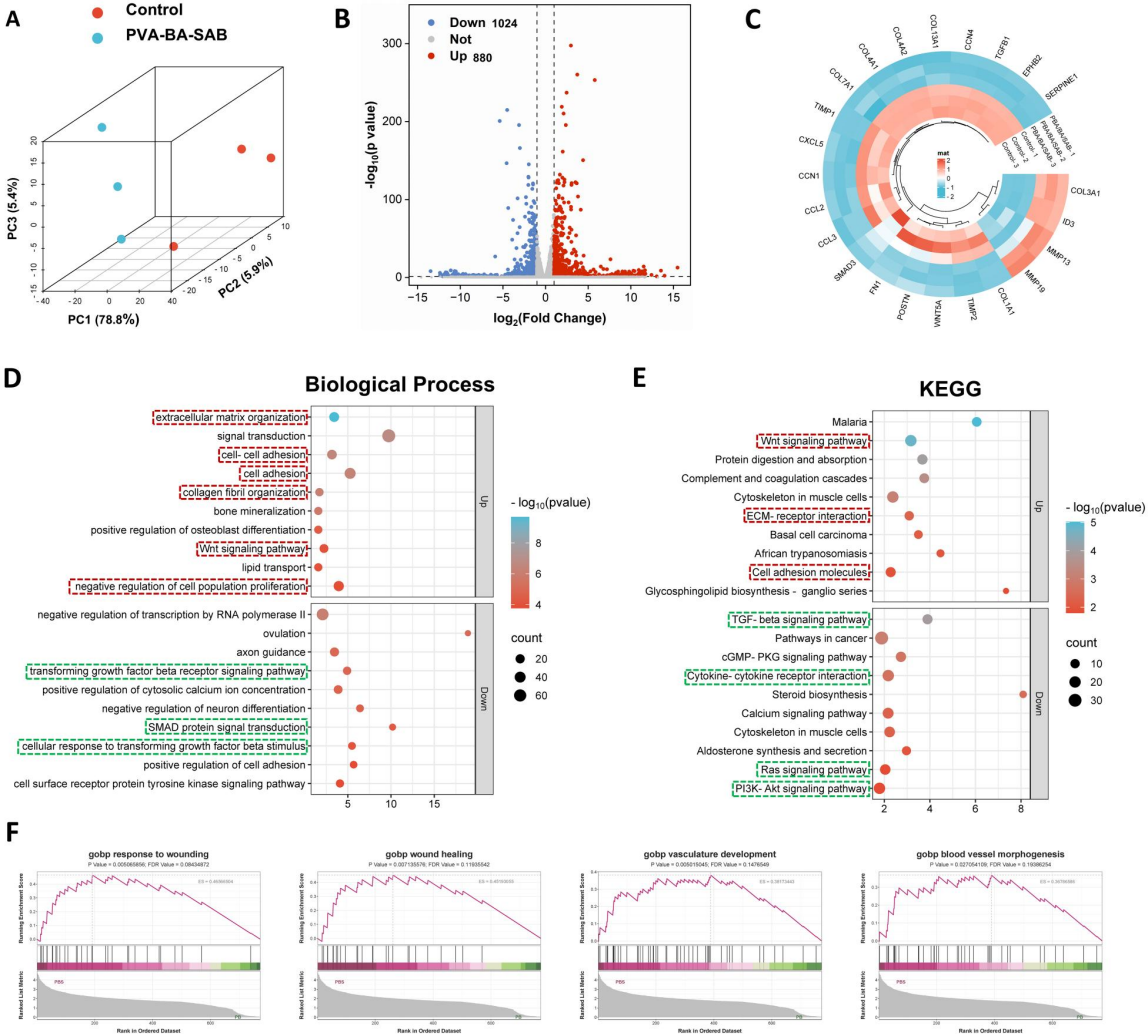
Mechanisms of PVA-BA-SAB hydrogel in inhibiting fibrosis. (**A**) Principal component analysis of all samples. (**B**) Volcano plot of the DEGs between the control group and the PVA-BA-SAB group. (**C**) Heatmap of DEGs related to fibrosis between the control group and the PVA-BA-SAB group. (**D**) GO enrichment analysis of DEGs between the control group and the PVA-BA-SAB group. (**E**) KEGG enrichment analysis of DEGs between the control group and the PVA-BA-SAB group. (**F**) GSEA enrichment analysis related to wound healing and angiogenesis between the control group and the PVA-BASAB group.

In order to comprehensively reveal the phenotypic transformation mechanisms of activated fibroblasts, we performed transcriptome sequencing on cells before and after treatment. The results of principal component analysis revealed a distinct separation between the two groups (Figure 4A). There was a total of 1904 DEGs, of which 880 were up-regulated and 1024 were down-regulated (Figure 4B). Figure 4C shows the expression level of fibrosis-related genes. Following treatment with PVA-BA-SAB hydrogel, the expression of fibrosis-inhibiting genes such as ID3/MMP9/MMP13 significantly increased, while fibrosis-promoting genes such as TGF-β, SMAD, FN1 and CCN family were down-regulated. In addition, the secretion levels of various collagens decreased except for COL3a1.

The up-regulated or down-regulated genes were, respectively, analyzed by GO and KEGG. The enrichment terms of Biological Process within GO analysis are shown in Figure 4D. Up-regulated genes were mainly enriched in ECM organization, cell adhesion, Wnt signaling pathway and negative regulation of cell population proliferation, while down-regulated genes were enriched in TGF-β receptor signaling pathway, SMAD protein signaling transduction and cellular response to TGF-β stimulus. The KEGG analysis results (Figure 4E) of up-regulated genes also showed enrichment of terms such as Wnt signaling pathway, ECM-receptor interaction, and cell adhesion molecules. Decreased genes were enriched in terms such as TGF-β signaling pathway, cytokine–cytokine receptor interaction, Ras signaling pathway, and PI3K-Akt signaling pathway. These results fully proved the anti-fibrotic ability of PVA-BA-SAB hydrogel by regulating the TGF-β signaling pathway.

In addition, in view of the initial role of fibroblasts in wound healing and angiogenesis, we performed Gene Set Enrichment Analysis (GSEA) analysis on GO terms such as response to wounding, wound healing, vasculature development, and blood vessel morphogenesis (Figure 4F). The results showed a clear directivity of these terms in the PVA-BA-SAB group. The enriched *P* values were all less than 0.05 and the FDR values were all less than 0.25, preliminarily proving the promoting healing and angiogenesis functions through PVA-BA-SAB treatment.

### Effect of PVA-BA-SAB hydrogel on wound healing

**Figure 5.**
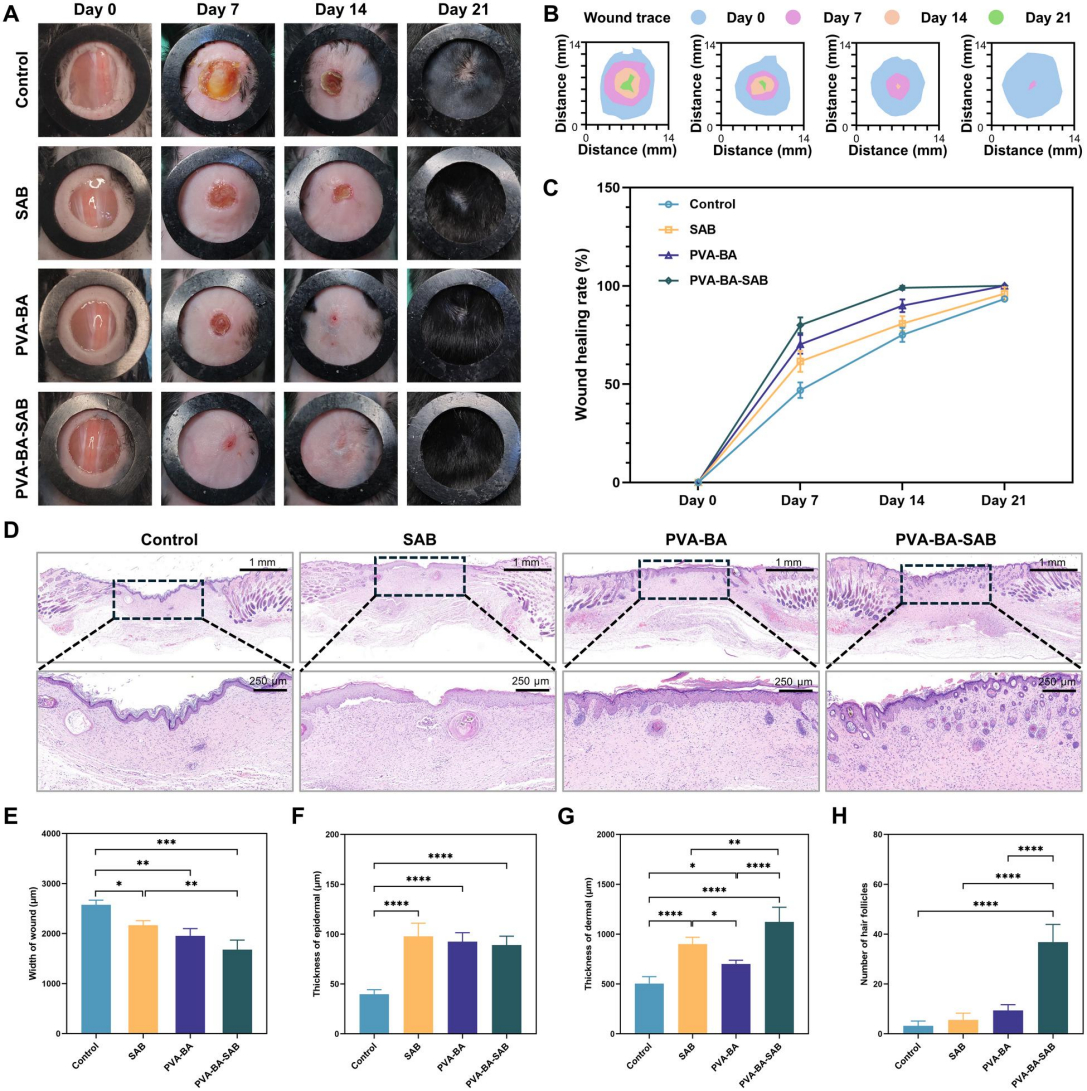
Effect of PVA-BA-SAB hydrogel on wound healing. (**A**) Representative photographs of wounds at different time points after various treatments. (**B**) Overlapping images of the extent of wound closure on days 0, 7, 14 and 21 in each group. (**C**) Quantification of the wound healing rate. (**D**) H&E staining images of wound tissues with different treatments on day 21. (**E–H**) Quantification of the width of wound, thickness of epidermal, thickness of dermal and number of hair follicles in wound tissues. *P < 0.05, **P < 0.01, ***P < 0.001 and ****P < 0.0001.

A full-thickness skin wound model was established on the backs of mice and treated with SAB solution, PVA-BA hydrogel or PVA-BA-SAB hydrogel to investigate the properties of the PVA-BA-SAB hydrogel in promoting wound healing. Figure 5A shows the representative optical images of gross wound contraction in the different groups on days 0, 7, 14 and 21. The PVA-BA-SAB group exhibited the most rapid wound closure compared to the other groups, demonstrating complete epithelialization by day 14. On day 21, the wound in the PVA-BA-SAB group was completely healed, with the most obvious evidence of hair regeneration. Figure 5B shows a discernible alteration in wound size in different groups. The quantitative values of the wound healing rate over different days are shown in Figure 5C. On day 7, the PVA-BA-SAB group showed a much faster wound healing rate (79.98 ± 3.26%) compared to the control (46.93 ± 3.21%), SAB (61.57 ± 4.35) and PVA-BA (70.22 ± 3.91%) groups. On day 14, the wound healing rate of PVA-BA-SAB group was 99.02 ± 0.81%, higher than the control (75.08 ± 2.89%), SAB (80.78 ± 3.12) and PVA-BA (89.89 ± 2.60%) groups.

To gain further insight into the therapeutic effects at the histological level, H&E staining was conducted on the regenerated skin tissues collected on day 21. The representative H&E staining images are shown in Figure 5D. The statistical results of wound width demonstrated the best promoting healing ability of the PVA-BA-SAB group (Figure 5E). The wounds in each group were all significantly epithelialized. The epidermal layer of the control group was obviously wrinkled, while the epidermal layers of the other three groups were flat. In the PVA-BA-SAB group, epidermal ridges could be clearly observed. The epidermal thickness of the control group (39.72 ± 4.10 μm) was significantly lower than other groups (Figure 5F), but there was a loose keratinized layer on the surface. The quantitative data of dermal thickness are shown in Figure 5G. Compared with the control group, the deposition thickness of the dermis layer increased. Only the dermal thickness of the PBA-BA-SAB group was similar to the surrounding uninjured skin. The number of regenerated hair follicles was further enumerated (Figure 5H). The PVA-BA-SAB group exhibited a density and maturity comparable to native hair follicles, regenerating alongside sebaceous glands.

### Evaluation of the anti-fibrotic effect of PVA-BA-SAB hydrogel

**Figure 6.**
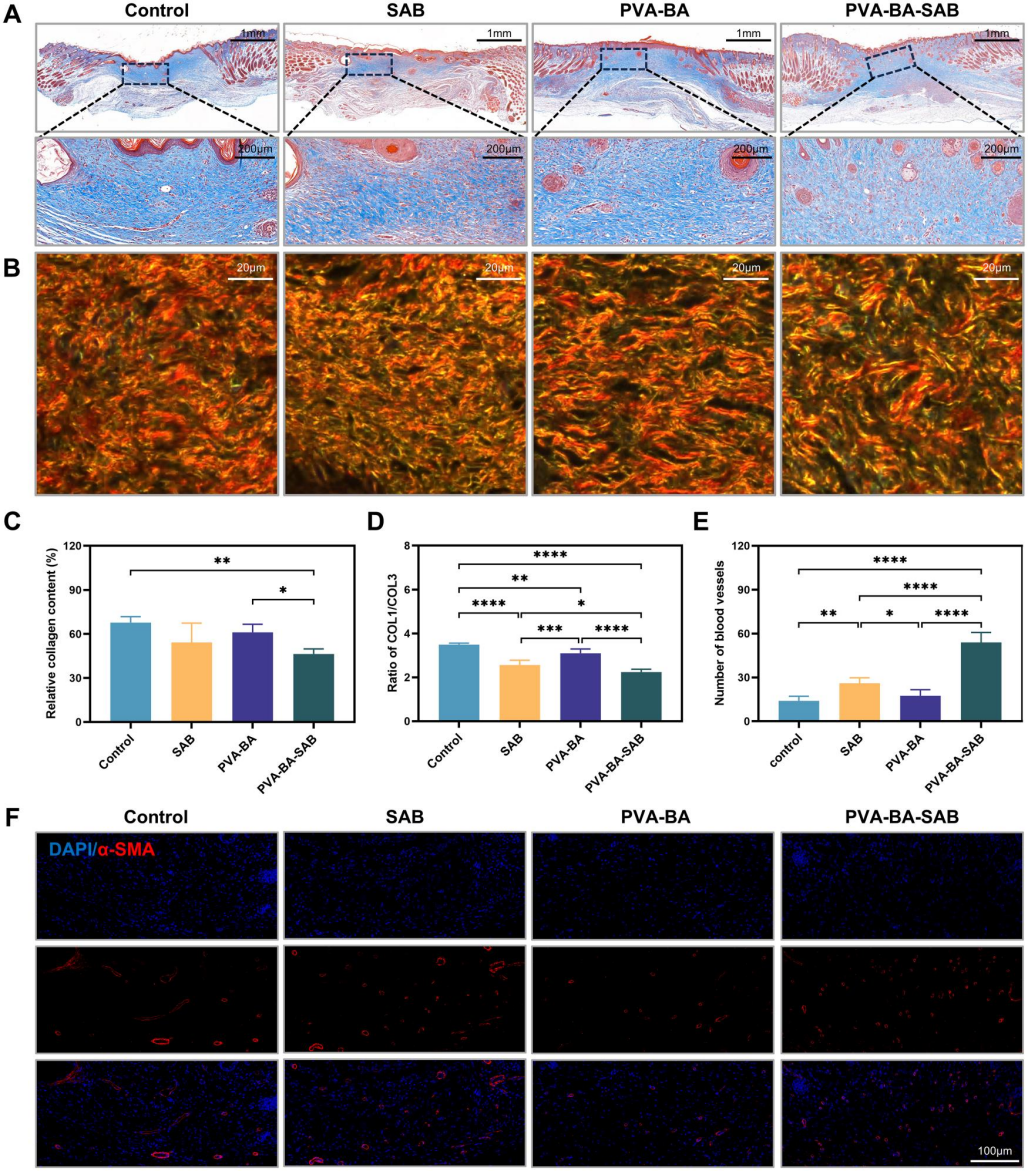
Evaluation of the anti-fibrotic effect of PVA-BA-SAB hydrogel. (**A**) Masson staining images of wound tissues with different treatments on day 21. (**B**) Picrosirius red staining images of wound tissues with different treatments on day 21. (**C**) Quantification of the relative collagen content in each group. (**D**) Quantification of the ratio of COL I/III in each group. (**E**) Quantification of the number of blood vessels in each group. (**F**) Immunohistochemical staining images of α-SMA in wound tissues with different treatments on day 21. *P < 0.05, **P < 0.01, ***P < 0.001 and ****P < 0.0001.

The level of collagen deposition in the wound area was evaluated using Masson staining (Figure 6A and B). The control group exhibited notable collagen deposition, with more thickened collagen bundles observed in the dermal region. The collagen distribution of the SAB group in different regions was obviously uneven. The PVA-BA group exhibited a similar collagen deposition with the control group, with less thickened collagen bundles. The collagen in the PVA-BA-SAB group was the least and uniform, approaching normal collagen deposition level and collagen morphology. The collagen arrangement and collagen type of the wound area were further evaluated through Sirius red staining (Figure 6C). Similar to the Masson staining, the wound area in the control group contained the highest amount of type I collagen, exhibiting a denser and coarser morphology. Collagen in the SAB group was distributed the most unevenly, while the PVA-BA group showed many thick collagen bundles. In the PVA-BA-SAB group, the thickened type I collagen was obviously reduced. Meanwhile, quantitative analysis (Figure 6D) showed that the ratio of COL I/III was significantly lower than the other three groups. The angiogenic potential of the hydrogels was evaluated by immunofluorescence staining of α-SMA, as shown in Figure 6E and F. The number of blood vessels in the SAB group was moderately elevated in comparison to the control and PVA-BA groups, yet it remained lower than the PVA-BA-SAB group. Small-diameter blood vessels with more uniform distribution were observed.

## Discussion

This study demonstrates the efficacy of a PVA-BA-based hydrogel featuring a dual-crosslinked network for the controlled release of SAB to inhibit fibrosis. This hydrogel exhibits excellent biocompatibility, mechanical properties and adhesive capacity, ensuring localized and sustained delivery of SAB. Such characteristics minimize systemic side effects while maximizing therapeutic efficacy at the injury site. *In vitro* experiments revealed significant anti-fibrotic activity, characterized by reduced expression of the TGF-β signaling pathway and activation of the Wnt signaling pathway, leading to the inhibition of fibroblast activation. *In vivo* studies further confirmed that this system effectively prevents excessive and uneven collagen deposition, optimizing collagen composition and creating an ideal regenerative microenvironment for the epidermis, hair follicles and blood vessels. Overall, the PVA-BA-SAB hydrogel significantly reduces wound fibrosis and enhances the quality of wound healing, providing a promising strategy for scar prevention.

The development of the PVA-BA-based dual-crosslinked network hydrogel as a controlled-release system for SAB represents a novel approach to managing scar formation. Not only does the hydrogel ensure sustained delivery of the active compound, but it also demonstrates impressive biocompatibility and mechanical properties, making it suitable for clinical applications, especially in areas prone to stretching or high activity. The effective closure of wounds with the PVA-BA hydrogel can prevent further injury and contamination. Although our cell experiments, utilizing an indirect contact model, showed no significant impact on fibroblast function from the hydrogel alone, in animal studies, the PVA-BA hydrogel was observed to promote wound healing, accelerate re-epithelialization, and regulate collagen deposition. By maintaining a moist microenvironment in the wound area, the hydrogel facilitates the migration and proliferation of epidermal cells, thereby accelerating wound closure [23, 24]. Additionally, its physical structure effectively inhibits wound contraction, reducing the risk of scar formation due to tension [25].

The application of SAB in scar inhibition and the exploration of its underlying mechanisms mark a significant advancement in wound healing research. Fibroblasts play a crucial role in this process, being transiently activated following injury [26, 27]. However, persistent excessive activation can lead to pathological scar formation [28, 29]. In our study, we pre-activated fibroblasts with TGF-β1 and investigated the controlled release of SAB from the PVA-BA-SAB hydrogel. Results indicated that within 48 hours, SAB was released steadily, without rapid diffusion in the initial hours. Given that the half-life of SAB is less than 3 hours [17], this controlled-release mechanism effectively prolongs the bioavailability of SAB. Notably, under acidic conditions (pH 5.5), the release of SAB increased by 13% due to the dissociation of borate ester bonds. During the early phases of injury, factors such as coagulation, inflammation, and the accumulation of lactic acid and carbon dioxide contribute to a reduced pH of the wound (typically 5–6) [30, 31]. The pH-responsive nature of the PVA-BA-SAB hydrogel ensures timely delivery of SAB, enhancing its effectiveness during the inflammatory response. Cell-based experiments further demonstrated that SAB can inhibit the proliferation and migration of activated fibroblasts while regulating collagen secretion, ultimately improving ECM deposition. This comprehensive regulatory effect on the radical biological behavior of over-activated fibroblasts enables fibroblasts to promote wound healing in a gentler way.

Our study also provides novel insights into the mechanisms by which SAB inhibits skin fibrosis through transcriptome sequencing. GO and KEGG analyses revealed that genes upregulated following treatment with the PVA-BA-SAB hydrogel were enriched in pathways related to ECM deposition, cell adhesion, and the Wnt signaling pathway, while the TGF-β signaling pathway was significantly downregulated. The Wnt signaling pathway is known to play a critical role in maintaining epidermal stem cells, promoting angiogenesis, and facilitating hair follicle development and regeneration [32, 33], whereas TGF-β is associated with pathological scar formation and Wnt pathway inhibition [34, 35]. Importantly, the use of SAB alone did not yield satisfactory therapeutic outcomes in animal models. Although re-epithelialization improved, the epidermal layer remained loose and detached from the dermis, with a sparse number of hair follicles. Additionally, while dermal thickness increased, collagen deposition was uneven. This inconsistency may stem from the heterogeneous distribution of SAB when applied topically. Furthermore, the rapid metabolism of SAB on the wound surface when used alone likely reduces its bioavailability, limiting its overall therapeutic efficacy.

When SAB is incorporated into the PVA-BA hydrogel, it functions as a sustained-release system, significantly prolonging the residence time of SAB at the wound site and ensuring uniform drug distribution. This innovative drug delivery system demonstrates remarkable regenerative efficiency. The formation of epidermal ridges—where the epidermis extends into the underlying connective tissue—highlights its potential to maintain epidermal stem cell function and prevent scar formation. In contrast, scar tissue typically lacks these vital epidermal ridges, resulting in dysfunctions such as increased susceptibility to peeling [36–38].

**Figure 7.**
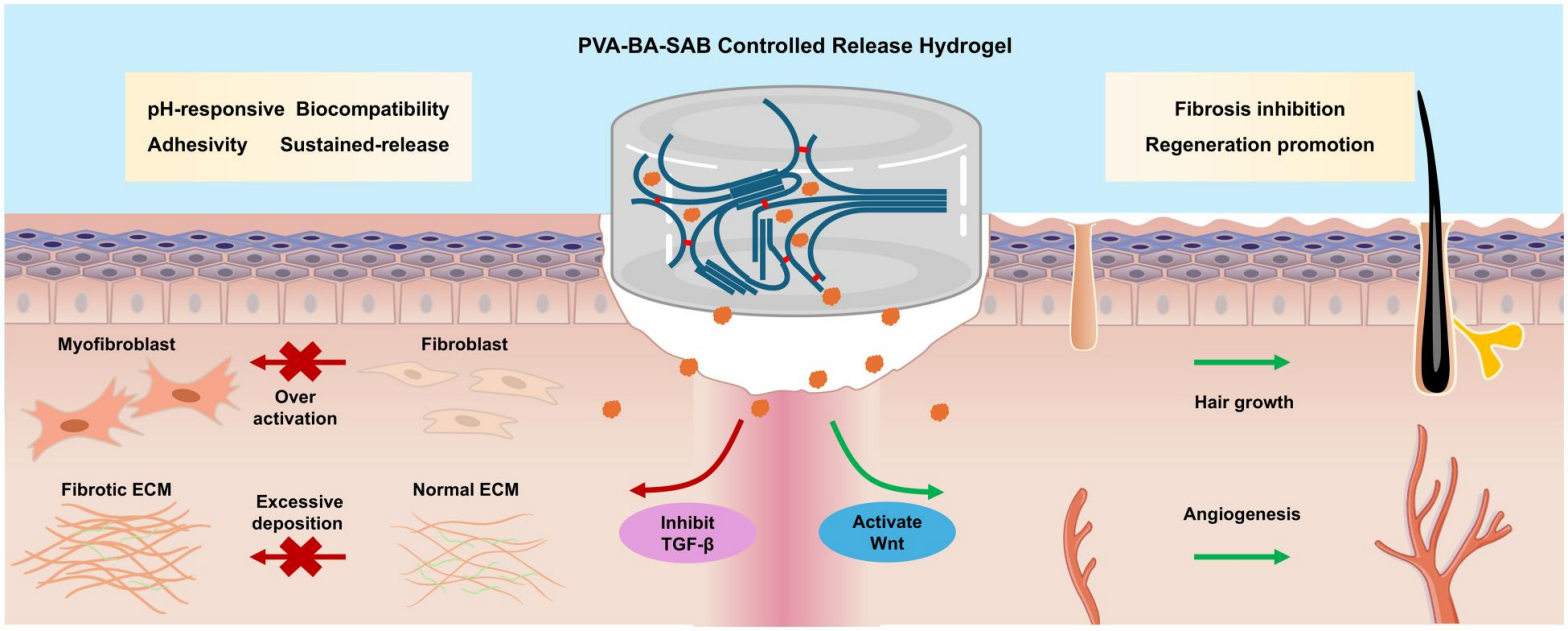
Schematic illustration of PVA-BA-SAB hydrogel for treatment of skin wound. PVA-BA-SAB controlled release hydrogel deliver SAB into the wound site consistently and evenly. The hydrogel effectively inhibits the TGF signaling pathway and activates the Wnt signaling pathway. It plays a vital role in preventing wound fibrosis by inhibiting over-activation of fibroblasts and excessive deposition of ECM. Meanwhile, the hydrogel enhances skin regeneration by promoting re-epithelialization, angiogenesis and hair growth.

Moreover, the treatment led to an increase in dermal thickness while significantly reducing the ratio of type I to type III collagen. This balanced collagen composition is critical for skin resilience and functionality [39, 40]. The regeneration of hair follicles accompanied by sebaceous glands, along with the development of small-diameter blood vessels, underscores the comprehensive recovery of skin function. These dual effects may be attributed to the antagonistic interaction between TGF-β and Wnt signaling pathways, which play pivotal roles in tissue regeneration [34, 41] (Figure 7). The PVA-BA-SAB hydrogel not only explores the previously unknown biological functions of SAB through controlled release but also sets a new standard in scar management and wound healing strategies.

In a novel approach to wound treatment, we have successfully utilized a pH-responsive hydrogel to deliver SAB, marking a significant advancement in the prevention of scarring and the restoration of skin function. This method is notable for its ability to modulate the balance between the TGF-β and Wnt signaling pathways, which plays a crucial role in the pharmacodynamics of SAB. These findings lay the groundwork for future research aimed at optimizing the PVA-BA-SAB hydrogel for clinical applications, with the potential to revolutionize treatment approaches in dermatology. This multifaceted innovation not only enhances therapeutic outcomes but also paves the way for improved patient care in scar management and skin regeneration.

## Conclusion

In conclusion, the PVA/BA-based dual-crosslinked network hydrogel loaded with SAB presents a novel approach to scar management. Its controlled release capabilities of SAB offer hope for improved outcomes in scar prevention and skin regeneration. Further clinical studies are warranted to validate these findings and advance this therapeutic strategy toward practical applications.

## Supplementary Material

rbaf002_Supplementary_Data

## Data Availability

The data supporting the findings of this study are available from the corresponding author upon reasonable request.
