# Supplementary material for: pH-responsive hydrogel with dual-crosslinked network of polyvinyl alcohol/boric acid for controlled release of salvianolic acid B: novel pro-regenerative mechanisms in scar inhibition and wound healing
Source: Regen Biomater. 2025 Jan 2;12:rbaf002. doi: 10.1093/rb/rbaf002 (PMC11785367; doi:10.1093/rb/rbaf002)
Supplement: rbaf002_Supplementary_Data [file rbaf002_supplementary_data.zip › 47c24_Supplementary File.docx]

**pH-Responsive Hydrogel with Dual-crosslinked Network of Polyvinyl Alcohol/Boric Acid for Controlled Release of Salvianolic Acid B: Novel Pro-Regenerative Mechanisms in Scar Inhibition and Wound Healing**

**Supplementary Figures**

**
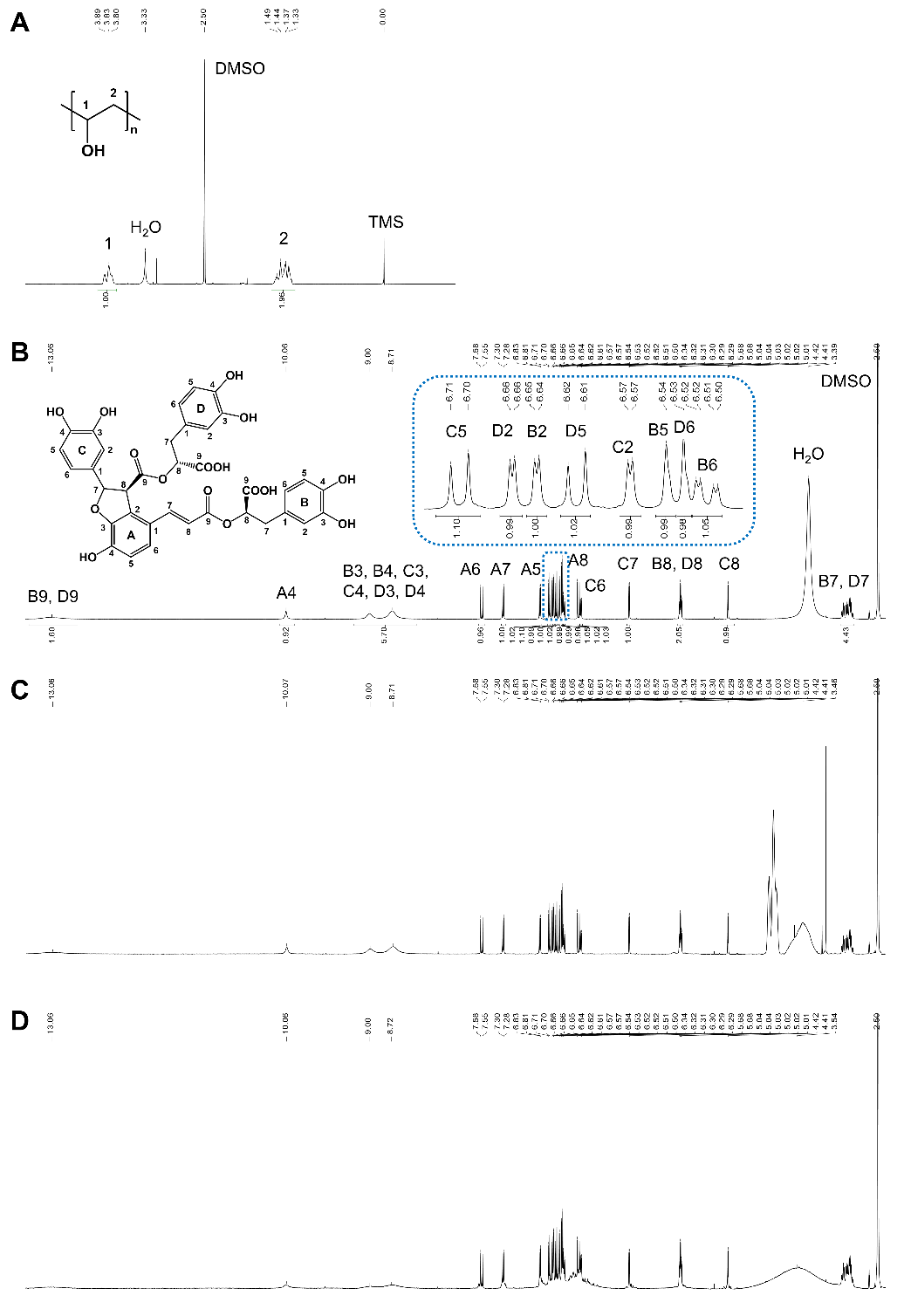
**

**Figure S1.** The NMR-H spectrum of PVA and SAB in different environments in DMSO-d_6_. (A) 300μl 10 mg/ml PVA + 300 μl DMSO-d_6_. (B) 300μl 10 mg/ml SAB + 300 μl DMSO-d_6_. (C) 300μl 10 mg/ml SAB + 300μl 10 mg/ml PVA. (D) 300μl 10 mg/ml SAB + 300μl10 mg/ml BA.

**
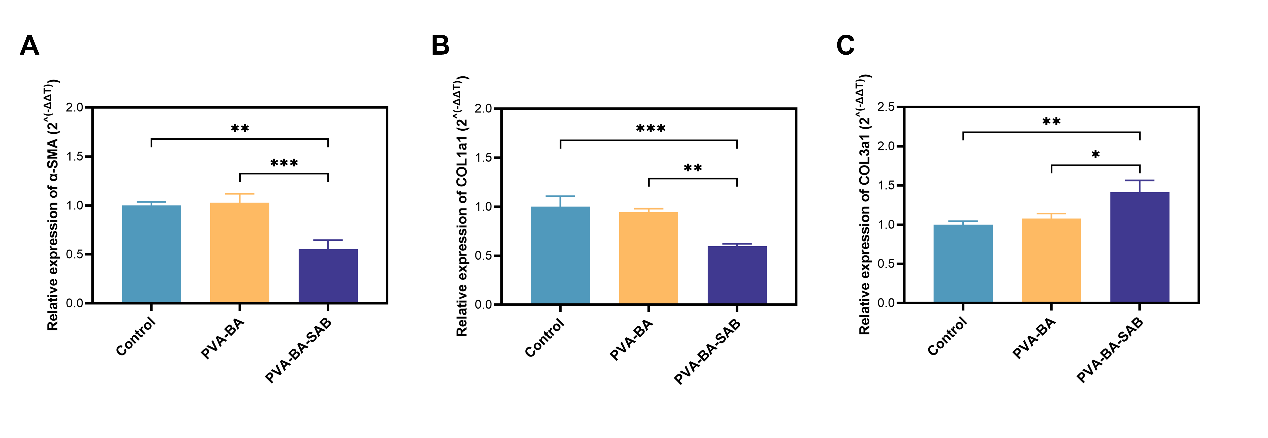
**

**Figure S2.** Anti-fibrosis effect of PVA-BA-SAB hydrogel evaluated by RT-qPCR. (A-C) Transcriptional expression of α-SMA, COL1a1 and COL3a1 in pre-actived HSF. ^*^*p* < 0.05, ^**^*p* < 0.01, and ^***^*p* < 0.001.

**Supplementary Tables**

| **Genes** | **Forward primer** | **Reverse primer** |
| --- | --- | --- |
| GAPDH | CTGACTTCAACAGCGACACC | GTGGTCCAGGGGTCTTACTC |
| TGFB1 | CAATTCCTGGCGATACCTCAG | GCACAACTCCGGTGACATCAA |
| ACTA2 | GTGTTGCCCCTGAAGAGCAT | GCTGGGACATTGAAAGTCTCA |
| SMAD2 | CCGACACACCGAGATCCTAAC | GAGGTGGCGTTTCTGGAATATAA |
| SMAD3 | GCGTGCGGCTCTACTACATC | GCACATTCGGGTCAACTGGTA |
| COL1A1 | GAGGGCCAAGACGAAGACATC | CAGATCACGTCATCGCACAAC |
| COL3A1 | GGAGCTGGCTACTTCTCGC | GGGAACATCCTCCTTCAACAG |

**Table S1.** The specific gene primers used for RT-qPCR.

| **Antibody** | **Vendor** | **Catalog No.** | **Host** | **Dilution** |
| --- | --- | --- | --- | --- |
| Anti-Ki67 | Abcam | ab16667 | Rabbit | 1:250 |
| α-SMA | Abcam | ab7817 | Mouse | 1:500 |
| Collagen I | Abcam | ab88147 | Mouse | 1:200 |
| Collagen III | Abcam | ab184993 | Rabbit | 1:100 |
| Goat anti-mouse IgG H&L  (Alexa Fluor 488) | Invitrogen | A-32723 | Goat | 1:500 |
| Goat anti-mouse IgG H&L  (Alexa Fluor 594) | Invitrogen | A-11005 | Goat | 1:500 |
| Goat anti-rabbit IgG H&L  (Alexa Fluor 594) | Invitrogen | A-11012 | Goat | 1:500 |
| Goat anti-rabbit IgG H&L  (Alexa Fluor 488) | Invitrogen | A-11008 | Goat | 1:500 |

**Table S2.** The antibodies used for immunofluorescent staining.
